# Comparison of general anesthesia and continuous intravenous sedation for electrochemotherapy of head and neck skin lesions

**DOI:** 10.3389/fonc.2022.1011721

**Published:** 2022-11-18

**Authors:** Janez Benedik, Barbara Ogorevc, Simona Kranjc Brezar, Maja Cemazar, Gregor Sersa, Ales Groselj

**Affiliations:** ^1^ Department of Anesthesiology and Perioperative Intensive Care Medicine, University Medical Centre Ljubljana, Ljubljana, Slovenia; ^2^ Faculty of Medicine, University of Ljubljana, Ljubljana, Slovenia; ^3^ Department of Experimental Oncology, Institute of Oncology Ljubljana, Ljubljana, Slovenia; ^4^ Faculty of Health Sciences, University of Primorska, Izola, Slovenia; ^5^ Faculty of Health Sciences, University of Ljubljana, Ljubljana, Slovenia; ^6^ Department of Otorhinolaryngology and Cervicofacial Surgery, University Medical Centre Ljubljana, Ljubljana, Slovenia

**Keywords:** sedation, general anesthesia, electrochemotherapy, head and neck skin lesions, bleomycin

## Abstract

**Background:**

Electrochemotherapy of cutaneous tumor nodules requires local or general anesthesia. For multiple and larger nodules, general anesthesia is recommended by standard operating procedures. The choice of general anesthesia is at the discretion of the treating center. Continuous intravenous sedation is also an option. Our study aimed to elucidate the tolerability, safety and possible advantages of continuous intravenous sedation in comparison to general anesthesia in patients undergoing electrochemotherapy.

**Patients and methods:**

In the prospective study, 27 patients undergoing electrochemotherapy were either under general anesthesia or under continuous intravenous sedation. Evaluated were different endpoints, such as feasibility and safety, duration of anesthesia and compliance with the patients.

**Results:**

Ten patients were treated under general anesthesia, and 17 patients were under continuous intravenous sedation. The comparison of the approaches indicated that continuous intravenous sedation required a lower overall dosage of propofol, a shorter duration of anesthesia, a shorter time to reach an Aldrete score >8, and greater satisfaction of the patients with the procedure compared to general anesthesia.

**Conclusion:**

The results indicate the feasibility and safety of continuous intravenous sedation for patients undergoing electrochemotherapy of cutaneous tumor nodules. This proved the preferred choice of anesthesia due to its shorter duration and better compliance with the patients compared to general anesthesia.

## Introduction

Electrochemotherapy (ECT) has been established as a local cancer therapy with proven antitumor efficacy, especially in the treatment of head and neck skin malignancies (1–3). During ECT, electric pulses are applied to the tumor to transiently increase cell membrane permeability to different cytotoxic agents, such as bleomycin and cisplatin (4, 5). The cytotoxic drugs used in ECT are given either intravenously or intratumorally (6). The applied electric pulses induce muscle contractions and therefore require pain control.

The updated Standard Operating Procedures for ECT propose the procedure to be performed under general or local anesthesia or continuous intravenous sedation (7). Local anesthesia is recommended for small and solitary nodules, whereas general anesthesia or sedation is recommended for large and/or multiple nodules (7). The majority of patients with head and neck malignancies are treated in general anesthesia (8–12). However, the choice of anesthesia is at the discretion of a specific institution and the preference of the anesthesiologist. The treatment of deep-seated tumors is obviously in the domain of general anesthesia (13–16). Nevertheless, the treatment of large and/or multiple cutaneous tumor nodules could also be performed with continuous intravenous sedation.

Currently, ECT is frequently used for the treatment of head and neck cutaneous and mucosal tumors (2, 3, 8, 17–20). Since most patients with head and neck malignancies are old with multiple comorbidities, careful anesthesiologic evaluation is crucial to avoid adverse effects related to the procedure whenever treatment is performed under general anesthesia or continuous intravenous sedation (1, 3, 17–19).

The present, single-institution clinical study aimed to evaluate the tolerability, safety and possible advantages of continuous intravenous sedation compared to general anesthesia in a group of patients with head and neck skin lesions treated with ECT.

## Patients and methods

### Patient selection

The study was conducted as a nonrandomized prospective study between April 2016 and April 2018 at the Department of Otorhinolaryngology and Cervicofacial Surgery, University Medical Centre Ljubljana, Slovenia. The selection of patients suitable for ECT was made in concordance with the inclusion and exclusion criteria listed in Standard Operating Prodecures for ECT (7, 21). The multidisciplinary head and neck tumor board confirmed the indications for ECT treatment and written informed consent was obtained from all included patients. The study protocol was approved by the National Medical Ethics Committee of the Republic of Slovenia (182/02/14 and 0120-132/2015-2).

Patients with multiple tumors or tumors greater than 1 cm in diameter were considered candidates for ECT under general anesthesia or continuous intravenous sedation. Before ECT, an anesthesiologist determined the type of anesthesia (general anesthesia or continuous intravenous sedation) for each patient according to the criteria listed below.

Criteria for patients selected for ECT under general anesthesia:

1. Refuse treatment under continuous intravenous sedation.
2. Expected difficulties with airway management – the need for endotracheal intubation (BMI>40, obstructive sleep apnea, tumors along the airway, Mallampati 3 or 4).
3. Expected longer duration of the procedure.

The criteria for performing ECT under continuous intravenous sedation:

1. There were no airway obstructions or difficulties with breathing.
2. Multiple systemic diseases (cardiovascular disease, diabetes mellitus, chronic kidney failure1^st^ and 2^nd^ degree).
3. The patient preferred to avoid general anesthesia.
4. Need for fast recovery and mobilization soon after the procedure.
5. Older patients with cognitive decline.
6. The American Society of Anesthesiologists (ASA) physical score was 1 to 3.

### Anesthesia technique

General anesthesia was performed with an intravenous bolus dose of propofol according to the patient’s body weight and age (1-2 mg/kg), and it was maintained by intravenous propofol infusion (3-4 mg/kg/h) or inhalational anesthesia (sevoflurane up to 1.5 vol %). Intraoperative and postoperative pain was relieved by administering an intravenous metamizole bolus dose (2.5 g), paracetamol intravenous bolus (1 g) and remifentanil infusion (0.1 – 0.3 µg/kg/min). The muscular relaxant rocuronium (0.45 – 0.6 mg/kg) was administered in all patients where ECT was performed under general anesthesia with endotracheal intubation.

Continuous intravenous sedation was performed with intravenous propofol infusion (1-2 mg/kg/h) or propofol intravenous bolus dose according to the patient’s body weight and age (1-1.5 mg/kg). Intraoperative and postoperative pain was relieved by administering an intravenous metamizole bolus dose (2.5 g), paracetamol intravenous bolus (1 g), remifentanil intravenous infusion (0.1-0.15 µg/kg/min) or fentanyl bolus dose 1-2 µg/kg. Muscular relaxants in patients with intravenous sedation were not administered.

The bispectral index (BIS) was used to monitor the depth of general anesthesia or intravenous sedation and intraoperative awareness during anesthesia. The BIS value was expected to be between 70 and 85 during continuous intravenous sedation and between 40 and 60 during general anesthesia. The BIS sensor was placed on a patient’s forehead before starting with the application of anesthetic drugs and removed after the BIS reached 85 or over and the patient was fully awake (22, 23). Monitoring the depth of anesthesia is very important during any procedure, as anesthesia that’s too deep can cause hemodynamic changes. Awareness during anesthesia is a very serious complication with potential long-term psychological sequelae such as anxiety and post-traumatic disorder. The BIS monitor is the first method that is FDA approved to assess the hypnotic effects of drugs. The bispectral index is a statistically based, empirically derived complex parameter. It is a weighted sum of several electroencephalographic subparameters, including a time domain, frequency domain, and high-order spectral subparameters. The BIS monitor provides a single dimensionless number, which ranges from 0 (equivalent to EEG silence) to 100. A BIS value between 40 and 60 indicates an appropriate level for general anesthesia, as recommended by the manufacturer. The BIS monitor thus gives the anesthetist an indication of how “deep” under anesthesia the patient is. The essence of BIS is to take a complex signal (the EEG), analyze it, and process the result into a single number. When a subject is awake, the cerebral cortex is very active, and the EEG reflects vigorous activity. When asleep or under general anesthesia, the pattern of activity changes.

Overall, there is a change from higher-frequency signals to lower-frequency signals (which can be shown by Fourier analysis), and there is a tendency for signal correlation from different parts of the cortex to become more random. The developers of the BIS monitor collected many (around 1000) EEG records from healthy adult volunteers at specific clinically important end-points and hypnotic drug concentrations. They then fitted bispectral and power spectral variables in a multivariate statistical model to produce the BIS index. As with other types of EEG analysis, the calculation algorithm that the BIS monitor uses is proprietary (22, 24).

### Procedure

ECT was performed according to the updated Standard Operating Procedures (7). BLM (Bleomycin medac; Medac, Wedel, Germany) was administered as an intravenous bolus injection in 2 minutes at a dose of 10000 IU/m^2^ to 15000 IU/m^2^ body surface area (1000 IU is equal to 1 mg of bleomycin activity). The electric pulses were applied 8 minutes after the BLM injection by needle row electrodes with fixed geometry (N-20-4B, IGEA, s.r.l., Carpi, Italy). Electric pulses were generated by a Cliniporator Pulse Generator (IGEA, s.r.l.). The electric pulses were applied several times to the tumor with repositioning of the electrodes to cover the whole tumor area, including the safety margins. The largest tumor diameter was measured with a caliper.

### Data collection

The clinical protocol was designed to obtain data necessary for the completion of the study. Demographic data and tumor characteristics were collected as a part of the InspECT protocol (25). The latest version of the ASA physical status classification system has been used to assess a patient’s preanesthesia medical comorbidities, predict perioperative risks and determine the safety and tolerability of continuous intravenous sedation or general anesthesia (26). The American Society of Anesthesiologists (ASA) Physical Status Classification System is a tool used in preparation for surgery to help predict risks in a given patient. The system uses a scale based on the patient’s medical history, the severity of known medical conditions, and current physical state to help predict if they can tolerate anesthesia and the conditions of surgery. The ASA Physical Status Classification System has been used for more than 60 years and was updated in 2019 to include additional disease examples.

The ASA Physical Status Classification System uses a scale from I to VI, with I being a healthy patient with minimal risks, to VI being a brain-dead patient with plans for organ donation (27).

The duration of anesthesia was measured from the application of anesthetic drugs until the complete vigilance of the patient (BIS > 85). Vital signs were measured by noninvasive blood pressure measurements, peripheral blood oxygenation (pulse oximetry), electrocardiography, end-tidal CO_2_ and ventilatory parameters during the entire perioperative period. Propofol bolus dose in mg, continuous propofol infusion in mg/kg/h and total propofol dosage in mg were registered for each patient. Furthermore, all the analgetic drugs (metamizole, paracetamol, remifentanil, fentanil) in standardized doses were preselected to ensure appropriate pain control.

The Ramsey Sedation Scale (RSS) was used in continuous intravenous sedation to assess the depth of sedation before, during and after the procedure. The Ramsay Sedation Scale (RSS) was the first scale to be defined for sedated patients and was designed as a test of rousability. The RSS scores sedation at six different levels (1 = Patient is anxious and agitated or restless, or both; 2 = Patient is cooperative, oriented and tranquil; 3 = Patient responds to commands only; 4 = Patient exhibits brisk response to a light glabellar tap or loud auditory stimulus; 5 = Patient exhibits a sluggish response to light glabellar tap or loud auditory stimulus; 6 = Patient exhibits no response), according to how rousable the patient is. It is an intuitively obvious scale and therefore lends itself to universal use, not only in the ICU, but wherever sedative drugs or narcotics are given. It can be added to the pain score and be considered the sixth vital sign (28).

The RSS score was measured before applying the anesthetic drug and after 5 and 10 min of sedation. During ECT procedures, the target RSS value was between 3 and 5 to assure moderate or deep sedation levels (29).

The evaluation of patient recovery time was measured with the Aldrete score based on the evaluation of vital signs and consciousness, and it was used to determine the time when a patient could safely leave the Post-Anaesthesia-Care Unit (PACU) and be transferred to the surgical ward. The patient was judged fit for discharge from the PACU when an Aldrete score > 8 was reached (30, 31).

The numeric pain scale (NPS) was used to assess pain before and after the procedure under continuous intravenous sedation and before and after general anesthesia. It was categorized into no pain = 0, mild pain = 1-3, moderate pain = 4-6, and severe pain = 7-10 (32).

Before leaving the PACU, a three-point satisfaction scale (very satisfied, satisfied or unsatisfied) was used to measure patients’ satisfaction with the procedure. Further details of the clinical study protocol and anesthesia technique are described in Supplement 1.

Preoxygenation with a 30% fraction of inspired oxygen was used to prevent hypoxemia during continuous intravenous sedation or endotracheal intubation in cases performed under general anesthesia (33, 34).

### Statistics

Continuous variables are presented as the mean value with standard error of the mean (SE), and categorical variables are presented as absolute numbers with percentages. Data were tested for normal distribution (D`Agostino&Pearson test). If data passed the normality test, the comparisons between groups were performed by the unpaired t-test (two-tailed). Significance was defined as p < 0.05. Data that did not pass the normality test are presented as the median and range: 25^th^ percentile - 75^th^ percentile, and the Mann−Whitney test was used for the comparison between groups. Categorical data were analyzed using Chi-square (and Fisher`s exact) (two-sided) test. Significance was defined as p < 0.05. Statistical analyses and graphical presentation of data were performed using GraphPad Prism 9.4.0 (673) (GraphPad Software, CA, US).

## Results

### Demographics and treatment groups

Overall, 27 patients (78% male and 22% female) with 113 nonmelanoma skin cancers in the head and neck region treated with ECT were included in the study. Except for one, all tumors were treatment naïve. The mean age of the group that underwent general anesthesia and continuous intravenous sedation was 69.1 and 74.8 years (p = 0.3580), respectively (**Table 1**).

**Table 1:** Patient demographics, procedure characteristics, patient compliance and short-term response.

	GENERAL ANESTHESIA	CONTINUOUS INTRAVENOUS SEDATION	P value
**Patients**	10 pts.	17 pts.	
**Gender**	80% male 20% female	76.5% male 23.5% female	0.8313
**Age**	69.1 ± 6.0 years	74.8 ± 3.0 years	0.3580
**Number of tumors**	73	40	
**Tumor diameter – (mean ± SE)**	30.0 ± 4.9 mm	21.2 ± 4.9 mm	0.0993
**ASA classification system score**	ASA 2 (20%) ASA 3 (80%)	ASA 2 (35%) ASA 3 (65%)	0.4202
**Propofol bolus (mean ± SE)**	121.0 ± 20.1 Infusion 1-6 mg/kg/h	73.5 ± 9.2 Infusion 1-2 mg/kg/h	0.0222
**Propofol total (mean ± SE)**	273.9 ± 50.6	82.7 ± 10.3	< 0.0001
**Duration of anesthesia (mean ± SE)**	68.9 ± 8.3 min	21.9 ± 3.1 min	< 0.0001
**ALDRETE > 8 (mean ± SE)**	6.2 ± 1.8 min	3.4 ± 0.8 min	0.1103
**NPS after anesthesia**	No pain (40.0%) Mild (20.0%) Moderate (40.0%)	No pain (47.1%) Mild (47.1%) Moderate (5.9%)	0.1683
**Satisfaction patient**	Very satisfied (60.0%) Satisfied (40.0%)	Very satisfied (64.7%) Satisfied (35.3%)	> 0.9999
**Satisfaction surgeon**	Very satisfied (70.0%) Satisfied (30.0%)	Very satisfied (76.5%) Satisfied (23.5)	> 0.9999
**Complications**	None (60%) Transient apnea (10%) Lumbar pain (30%)	None (47.0%) Transient apnea (47.0%) Lumbar pain (5.9%)	0.0717
Tumor response 2 months after therapy*	CR (78.1%)	CR (90.0%)	0.1299
	PR (21.9%)	PR (10.0%)	

P value: comparison between the general anesthesia and continuous intravenous sedation groups; * tumor response assessed according to RECIST criteria; CR, complete response; PR, partial response.

**Figure 1:**
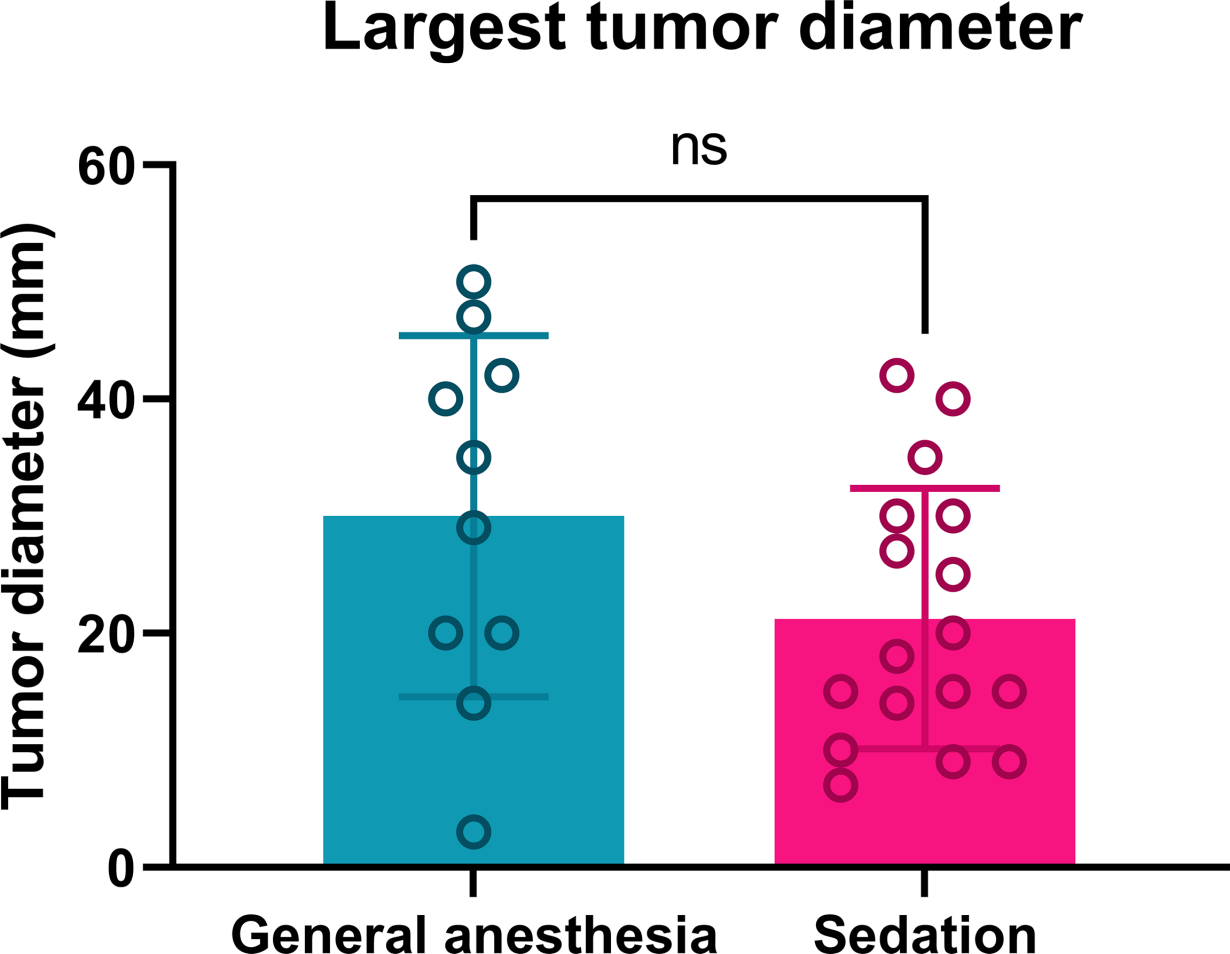
Tumor diameters of patients treated by ECT under general anesthesia or continuous intravenous sedation. Ns: nonsignificant; p = 0.0993.

The largest diameters of the treated tumors in both treatment groups were similar (**Figure 1**). General anesthesia was performed in 10 patients (8 male, 2 female). In this group, ECT was performed in 73 tumors, with a mean largest diameter of 30.0 mm, and four patients had multiple tumors. Continuous intravenous sedation was used in 17 patients (13 male, 4 female), with ECT performed in 40 tumors, with a mean largest diameter of 21.2 mm. Six patients had multiple tumors (**Table 1**). There was no significant difference in gender distribution in the group of general anesthesia and continuous intravenous sedation (p = 0.8313). According to Standard Operating Procedures, all the patients were treated by ECT 8 minutes after *i.v.* BLM injection (7). Both the general anesthesia group and the group treated with continuous intravenous sedation were well matched regarding the demographics and tumor characteristics.

The physical status of patients before anesthesia was scored by the ASA classification system. The group treated in general anesthesia had a higher percentage of ASA 3 (80%) patients than the group treated in continuous intravenous sedation (65%) (p = 0.4202) (**Table 1**). All ASA 3 patients had two or more systemic diseases (arterial hypertension, chronic atrial fibrillation on therapy with anticoagulant drugs, previous cardiac events, diabetes mellitus, pulmonary diseases, chronic kidney failure 1^st^ and 2^nd^ degree).

### Consumption of anesthetics

**Figure 2:**
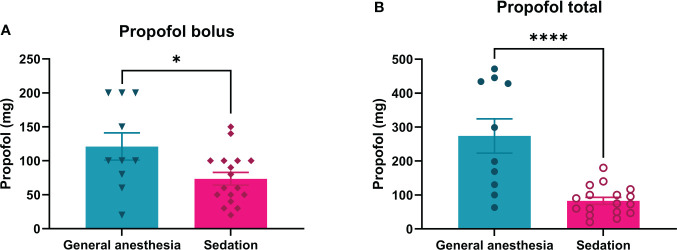
Consumption of propofol during ECT treatment of patients under general anesthesia or continuous intravenous sedation (Bolus: A; Propofol total: B). For continuous intravenous sedation of patients for ECT, less bolus and total propofol are needed than for general anesthesia. *p= 0.0222 and ****p= <0.0001.

The average loading dosage of the propofol bolus used during continuous intravenous sedation was significantly lower (73.5 ± 9.2 mg) than that used in the general anesthesia group (121.0 ± 20.1 mg; p = 0.0222) (**Figure 2A**). Continuous intravenous sedation was maintained with continuous intravenous infusion of 1-2 mg/kg/h of propofol, whereas under general anesthesia, continuous intravenous infusion of propofol was 1-6 mg/kg/h (**Table 1**). The mean value of total propofol used during continuous intravenous sedation was significantly lower (82.7 ± 10.3 mg) compared to the general anesthesia group (274.0 ± 50.6 mg; p < 0.0001) (**Table 1**; **Figure 2B**).

### Procedure characteristics

**Figure 3:**
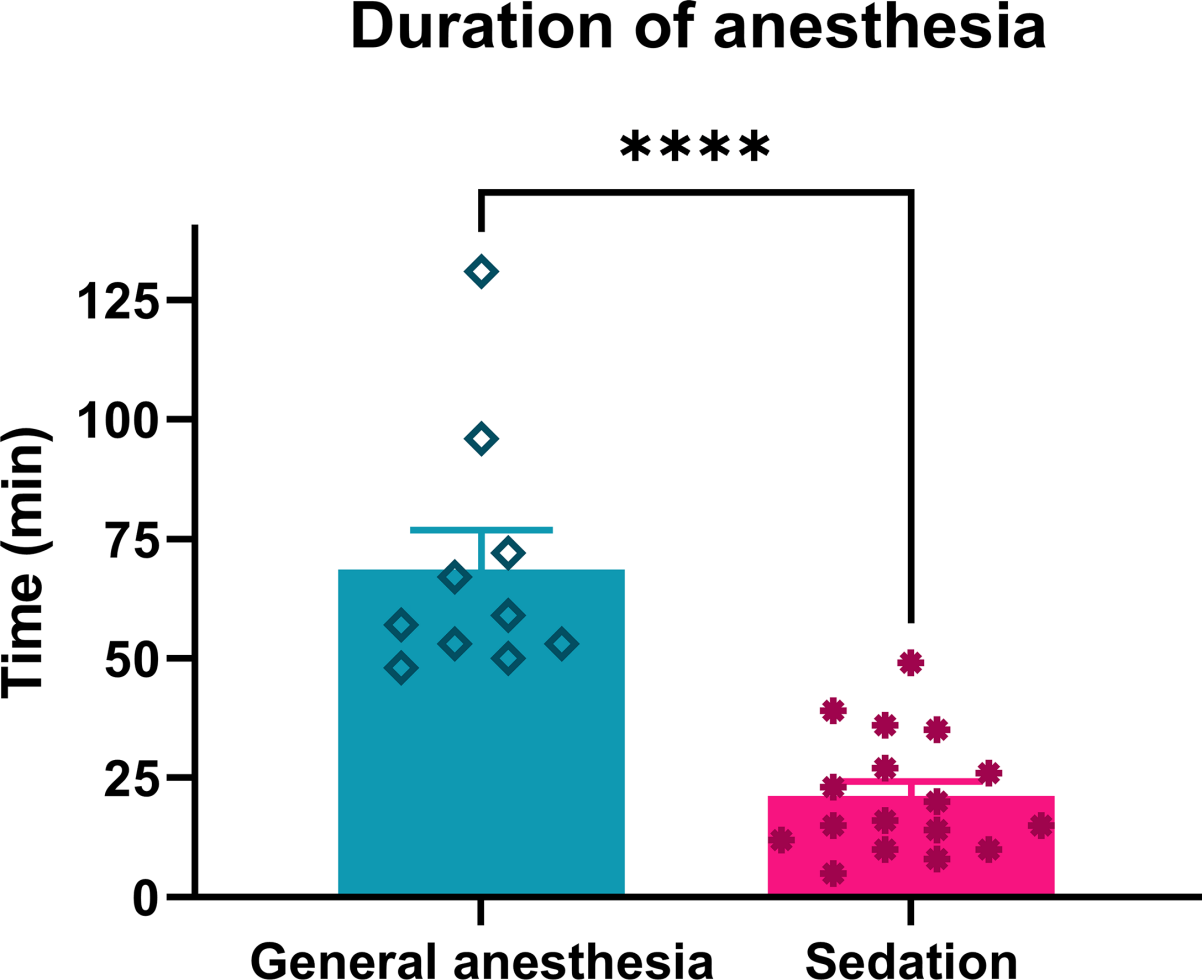
Duration of anesthesia in patients treated with ECT performed under general anesthesia or continuous intravenous sedation. ****p < 0.0001.

The anesthesia duration under continuous intravenous sedation was significantly shorter than that under general anesthesia (p = 0.0001) (**Table 1**; **Figure 3**).

**Figure 4:**
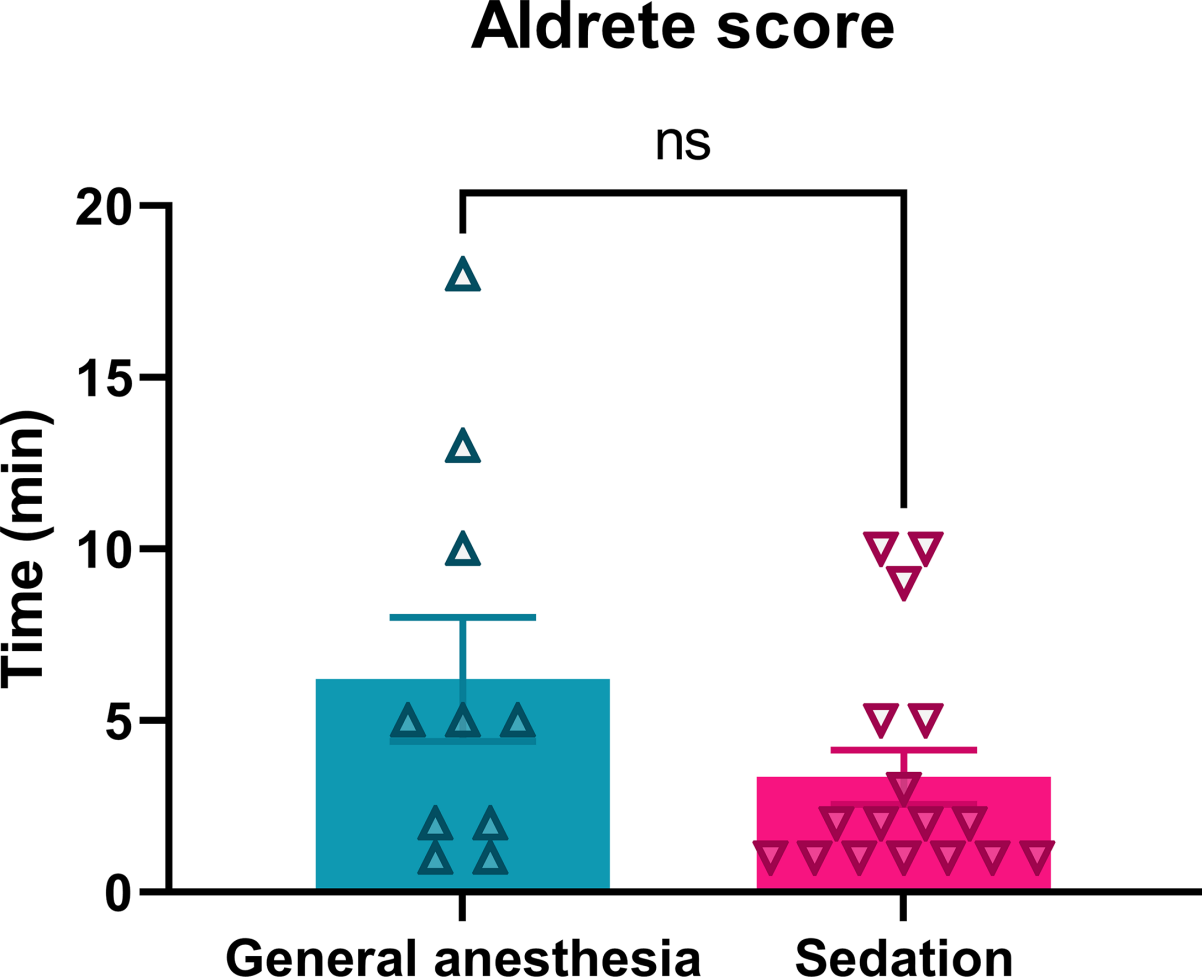
Recovery of patients treated with ECT performed under general or sedation procedures. Ns, nonsignificant, p = 0.1103.

The recovery of patients after anesthesia was faster after continuous intravenous sedation than after general anesthesia. The patients who underwent continuous intravenous sedation achieved an Aldrete score > 8 in the average time of 3.4 ± 0.8 min, while in the general anesthesia group, an Aldrete score > 8 was obtained in an average time of 6.2 ± 1.8 min, which was not significantly prolonged (p = 0.1103) (**Table 1**; **Figure 4**).

### Patient compliance

After the procedure, the patients were asked about the pain and discomfort. In the general anesthesia group, the numerical pain score (NPS) score was 3 or more in 60% of the patients. In the continuous intravenous sedation group, the NPS score after the procedure was 3 or more in 53% of the patients. There was no difference in NPS score between the patients in the general anesthesia group and the continous intravenous sedation group (p = 0.1683). Patients mainly complained of lumbar pain or pain caused by body positioning during the operation (**Table 1**).

In the PACU, patients were asked about their satisfaction with the procedure and general anesthesia/sedation (very satisfied, satisfied, not satisfied). Generally, patients were satisfied and very satisfied with the procedure and selected anesthesia. Patients who underwent continuous intravenous sedation were defined to be very satisfied after the procedure under sedation in 64.7%, while 60.0% of patients in the general anesthesia group were very satisfied (p > 0.9999) (**Table 1**). Additionally, the surgeons who performed ECT were very satisfied with general intravenous sedation (70%) as were with general anesthesia (76.5%) (p > 0.9999) (**Table 1**).

Complications during general anesthesia and continuous intravenous sedation were few. The most common expected event during continuous intravenous sedation was short episodes of transient apnea in 47% of patients. Otherwise, there were no significant adverse effects during continuous intravenous sedation and general anesthesia. The complications during and after ECT treatment in general anesthesia compared to the continuous intravenous sedation group were statistically nonsignificant (0.0717) (**Table 1**).

### Short-term local response

ECT proved to be effective similarly in patient groups of general anesthesia and continuous intravenous sedation (p = 0.1299). Two months after therapy a complete response (CR) rate of 78% and partial response (PR) rate 21.9% in the group of general anesthesia and CR rate of 90% and PR rate of 10% in the group of continuous intravenous sedation was observed (**Table 1**).

## Discussion

To the best of our knowledge, this is the first clinical study evaluating the tolerability, safety and possible advantages of continuous intravenous sedation in comparison to general anesthesia in patients treated with ECT. The results of our research have shown significant differences between both types of anesthesia. ECT could be performed in local anesthesia, continuous intravenous sedation and general anesthesia (21), but there are no unified standard anesthesia protocols for ECT. Every department where ECT is performed has its own protocols with different preferences for chosen anesthesia. In our institution, the preferred choice of anesthesia for ECT is continuous intravenous sedation. The advantages of performing ECT in sedation are avoidance of muscular relaxants, no need for endotracheal intubation and a short-lasting procedure. Thus, the usage of converting drugs such as sugammadex is not needed (35, 36).

The most important and evident difference is the dose of propofol used during anesthesia. In continuous intravenous sedation, we administered almost 40% less propofol (mg/kg) per person than in general anesthesia. Considering that the most of the patients were older than 65 years and had multiple comorbidities, a lower dose of propofol is beneficial. Propofol, like any other anesthetic, has side effects. In larger doses, as is bolus administration during the induction in general anesthesia, it is often associated with pain, apnea, hypotension, and rarely thrombophlebitis when injected intravenously (37). In contrast, with sedation, the slow onset with continuous intravenous infusion is less painful and more pleasant with fewer side effects. With careful titration of propofol, apnea during sedation can be avoided in most patients. With a lower overall dosage of propofol, we can prevent significant hypotension, especially in patients with cardiac diseases and arterial hypertension. Furthermore, propofol alone provides good or acceptable myorelaxation. This is especially important for optimal conditions since ECT can cause muscular contraction (38, 39).

According to Standard Operating Procedures, ECT can also be performed in local anesthesia. However, care must be taken when ECT is performed for multiple lesions or more extensive lesions. In these situations, the chance of a local anesthetic overdose is higher, especially if optimal operating conditions and painless procedures for the patients should be obtained. Most of the patients treated with ECT are old; therefore, they are also more prone to local anesthetic systemic toxicity (3, 19, 40). Relevant comorbidities that are known to increase the risks of local anesthetic systemic toxicity include age, low body mass index and coronary artery disease (41). The rate of disappearance of, e.g., lidocaine from blood, has been prolonged in patients with congestive heart failure, decreased hepatic blood flow and impaired liver enzymes. Lidocaine half-life is also significantly prolonged in patients over the age of 61 (40, 42).

Disorientation and drowsiness are two of the most common signs of neurotoxicity of local anesthetics. Elderly patients are at higher risk for potential toxicity of local anesthetics due to prolonged half-time, possibly presented with larger and multiple lesions and therefore higher doses of local anesthetic drugs are administered. Thus, preexisting cognitive decline and early signs of dementia can worsen after a significant dose of local anesthetic drugs. In addition, cardiovascular diseases are common among the elderly population who will undergo ECT. The main cardiovascular mechanism of local anesthetic toxicity is the blockade of cardiac sodium channels leading to negative inotropy and arrhythmia, which should be avoided (40, 43).

During the procedure under local anesthesia, patients are awake, and at least some are anxious and feel uncomfortable on the operating table. Consequently, unintentional movements can disrupt the procedure. In addition, ECT causes muscular contractions, which are unpleasant. This should be avoided since a patient should feel little or no discomfort during the procedure. Administration of local anesthetics can also be very painful because of the richness of innervation in the head and neck area (44–46).

The duration of anesthesia and the procedure was significantly shorter when continuous intravenous sedation was performed compared to the procedure peformed in general anesthesia. Thus, recovery and mobilization are faster compared to general anesthesia. The duration of anesthesia was not related to the number and size of treated lesions, since ECT is a short procedure. No statistically significant differences were observed between the groups regarding the size of the lesions. The pain was present mostly after the procedure and was not related to the procedure. The main complaint was lumbar pain due to body position. No statistically significant differences were observed regarding patient satisfaction with procedure between the groups.

Complications during general anesthesia and continuous intravenous sedation were few. The most common complication during continuous intravenous sedation was short episodes of transient apnea in 47% of patients. Transient apnea could be avoided with careful and gradual intravenous titration of propofol. In the general anesthesia group, complications were not recorded. In both groups, patients complained of lumbar pain after the operation due to body position during the procedure.

Adequate preoxygenation is crucial before sedation or endotracheal intubation to avoid hypoxemia. It is important to emphasize that we safely use preoxygenation with a maximum of 30% inspired oxygen to reduce the risk of BLM-associated lung toxicity, as is recommended in SOP (7).

Some might speculate that general anesthesia ensured optimal patient compliance, consequently allowing clinicians to apply electrodes deeper and in a broader field. However, our study demonstrates that ECT can be efficiently performed in sedation. Short-term evaluation of tumor response after ECT under sedation demonstrated the comparable rate of CR (90%) and PR (10%) to general anesthesia (rate CR of 78% and rate PR of 22%). The main advantage is the patient’s fast recovery and safer procedure since the dose of anesthetic drugs is smaller.

We are aware that our study has some limitations. For instance, one anesthesiologist provided both types of anesthesia. Some training and experience with accurately designed protocols are needed for adequate and safe anesthesia under continuous intravenous sedation. In different institutions, the propofol dosage or even the choice of anesthetic drugs might vary. Consequently, our protocol for continuous intravenous sedation during ECT is difficult to apply to all institutions where ECT is performed.

## Conclusions

The majority of patients treated with ECT are old with several comorbidities. Thus, a careful anesthesiology approach is mandatory to avoid complications related to anesthesia. Our study demonstrated that ECT could be safely performed under continuous intravenous sedation. A lower dosage of propofol during continuous intravenous sedation is beneficial in patients with advanced age, multiple systemic diseases and cognitive decline. Faster recovery time enables dismission from the hospital on the same day. Complications were few regardless of the type of anesthesia; transient apnea during continuous intravenous sedation could be avoided with careful, gradual propofol titration.

## Data availability statement

The raw data supporting the conclusions of this article will be made available by the authors, without undue reservation.

## Ethics statement

The studies involving human participants were reviewed and approved by National Medical Ethics Committee of the Republic of Slovenia (182/02/14 and 0120-132/2015-2). The patients/participants provided their written informed consent to participate in this study.

## Author contributions

Conceptualization: JB, GS, AG. Methodology, data acquisition and analysis: JB, BO, SK. Writing: JB, GS, AG, SK. Review and editing: AG, GS, MC and SK. All authors contributed to the article and approved the submitted version.

## Funding

This work was financially supported by the state budget by the Slovenian Research Agency, program no. P3-0003 and P3-0307.

## Conflict of interest

The authors declare that the research was conducted in the absence of any commercial or financial relationships that could be construed as a potential conflict of interest.

## Publisher’s note

All claims expressed in this article are solely those of the authors and do not necessarily represent those of their affiliated organizations, or those of the publisher, the editors and the reviewers. Any product that may be evaluated in this article, or claim that may be made by its manufacturer, is not guaranteed or endorsed by the publisher.
