# Supplementary material for: Comparison of general anesthesia and continuous intravenous sedation for electrochemotherapy of head and neck skin lesions
Source: Front Oncol. 2022 Nov 18;12:1011721. doi: 10.3389/fonc.2022.1011721 (PMC9717680; doi:10.3389/fonc.2022.1011721)
Supplement: Supplementary file 1 [file DataSheet_1.docx]

**Brief Research Report**

Comparison of general anesthesia and continuous intravenous sedation for electrochemotherapy of head and neck skin lesions

Janez Benedik^1,2^, Barbara Ogorevc^1^, Simona Kranjc Brezar^2,3^, Maja Cemazar^3,4^, Gregor Sersa^3,5^, Ales Groselj^2,6^

^1^Department of Anesthesiology and Perioperative Intensive Care Medicine, University Medical Centre Ljubljana, Ljubljana, Slovenia

^2^Faculty of Medicine, University of Ljubljana, Ljubljana, Slovenia

^3^Department of Experimental Oncology, Institute of Oncology Ljubljana, Ljubljana, Slovenia

^4^Faculty of Health Sciences, University of Primorska, Izola, Slovenia

^5^Faculty of Health Sciences, University of Ljubljana, Ljubljana, Slovenia

^6^Department of Otorhinolaryngology and Cervicofacial Surgery, University Medical Centre Ljubljana, Ljubljana, Slovenia

*** Correspondence:**Ales Groselj, Department of Otorhinolaryngology and Cervicofacial Surgery, University Medical Centre Ljubljana, Ljubljana, Slovenia, e-mail ales.groselj@kclj.si; Gregor Sersa, Department of Experimental Oncology, Institute of Oncology Ljubljana, Ljubljana, Slovenia, e-mail: gsersa@onko-i.si

**Standard Operating Procedures for continuous intravenous sedation**

# Purpose and scope

The purpose of the Standard Operating Procedures (SOPs) of the Clinical Department of Anesthesiology and Intensive Care of the Operating Disciplines is to standardize anesthetic procedures and thereby improve quality and safety.

# Procedure

## Specific features, problems and possible complications of surgery

The choice of the patient to undergo electrochemotherapy under sedation is important, contraindications include:

1. The patient refuses the procedure under sedation

2. Anticipated airway problems (BMI > 40, obstructive sleep apnoea, tumors in the airway, Mallampati airway assessment 3 and 4)

3. Expected prolonged intervention time

4. Inability of the patient to lie still (chronic cough, mental retardation, dementia, anxiety disorders,...)

The choice of anesthetic technique is intended to allow the patient to recover quickly, with fewer complications from anesthesia and a short hospital stay.

## Premedication

The patient does not receive premedication on the ward before sedation.

## Preparation of the work site

Anesthetic machines and drugs are prepared by a medical technician/nurse and checked by a doctor. 1 propofol pump is needed (2 pumps in case of general anesthesia; propofol and remifentanil).

Standard patient monitoring: non-invasive arterial blood pressure measurement, electrocardiogram (ECG), pulse oximetry, capnometry, monitoring the depth of sedation/anesthesia (BIS).

The patient is positioned comfortably to minimize the possibility of movement during the procedure.

## Type of anesthesia

**Preparation of the patient's position**

The patient is lying on his/her back with both arms by the side of the body, the head is away from the anesthetic machine, and difficult to reach during the procedure. A good intravenous route is established, if possible on the right arm; the anesthetic machine and the anesthetic trolley are also on the patient's right side. We also need an intravenous line in the leg where the oncologists inject the chemotherapeutic agent (bleomycin).

**General anesthesia (in case of contraindications to sedation)**

Anesthetic: propofol (1-2 mg/kg), maintenance by *i.v.* infusion (3-4 mg/kg/h) or inhalation with sevoflurane up to 1.5 volume %.

Analgesics: remifentanil infusion (0.2 – 0.3 μg/kg/min), paracetamol 1 g, metamizol 2.5 g.

Muscle relaxant: rocuronium (0.45 – 0.6 mg/kg)

Patients are intubated and ventilated in a controlled manner. Eyes well protected. Monitor depth of anesthesia with BIS (value 30 - 40).

**Sedation:**

Anesthetic: propofol in an initial small bolus (0.5-1.5 mg/kg *i.v.*) and/or propofol *i.v.* infusion (1-2 mg/kg/h).

Analgesics: paracetamol *i.v.* and/or metamizol *i.v.* in bolus, remifentanil / fentanyl *i.v.*

The most commonly used combination: is propofol in bolus (0.5-1.5 mg/kg *i.v.*), then remifentanil in *i.v.* infusion (0.1 - 0.2 μg/kg/min), fentanyl 1-2 μg/kg, paracetamol 1g *i.v.*, metamizol 2.5 g *i.v*..

Protect the eyes. Monitor depth of sedation with BIS (70 - 85).

**Postoperative pain relief**

Postoperative pain is mild, *i.v.* paracetamol 1 g or metamizol 2.5 g is sufficient.

**Postoperative monitoring**

The patient is transferred to the EPO, NIBP, O_2_ saturation, and ECG are monitored, he/she may be discharged when the Aldrete score is > 8 points.
